# Reliability of Thyroid Imaging Reporting and Data System (TI-RADS), and ultrasonographic classification of the American Thyroid Association (ATA) in differentiating benign from malignant thyroid nodules

**DOI:** 10.20945/2359-3997000000018

**Published:** 2018-03-23

**Authors:** Bruno Mussoi de Macedo, Rogério F. Izquierdo, Lenara Golbert, Erika L. Souza Meyer

**Affiliations:** 1 Universidade Federal de Ciências da Saúde de Porto Alegre Universidade Federal de Ciências da Saúde de Porto Alegre Endocrine Division Thyroid Section Porto Alegre RS Brazil Thyroid Section, Endocrine Division, Irmandade da Santa Casa de Misericórdia de Porto Alegre, Universidade Federal de Ciências da Saúde de Porto Alegre (UFCSPA), Porto Alegre, RS, Brazil

**Keywords:** Thyroid nodules, US patterns, thyroid cancer

## Abstract

**Objective:**

Ultrasonography (US) is the best diagnostic tool for initial assessment of thyroid nodule. Recently, data reporting systems for thyroid lesions, such as the Thyroid Imaging Reporting and Data System (TI-RADS) and American Thyroid Association (ATA), which stratifies the risk for malignancy, have demonstrated good performance in differentiating malignant thyroid nodules. The purpose of this study is to determine the reliability of both data reporting systems in predicting thyroid malignancy in a tertiary care hospital.

**Materials and methods:**

We evaluated 195 thyroid nodules using modified TI-RADS and ATA risk stratification. The results were compared to the cyto-pathology analysis. Histopathological results were available for 45 cases after surgery, which is considered the golden standard for diagnosis of thyroid cancer.

**Results:**

When compared with cytological results, sensitivity, specificity, negative predictive value (NPV), and accuracy were 100, 61.1, 100, and 63%, respectively, for TI-RADS; and 100, 75, 100, and 76%, respectively, for ATA. When compared with histopathological results, sensitivity, specificity, NPV, and accuracy were 90, 51.4, 94.7, and 60% respectively, for TI-RADS; and 100, 60, 100, and 68%, respectively, for ATA. All patients with malignant nodules were classified in the categories 4 or 5 of TI-RADS and in the intermediate or high suspicion risk according to the ATA system.

**Conclusion:**

Both TI-RADS and the ATA guidelines have high sensitivity and NPV for the diagnosis of thyroid carcinoma. These systems are feasible for clinical application, allowing to better select patients to undergo fine-needle aspiration biopsies.

## INTRODUCTION

Thyroid nodules are a common finding within the general population, and their detection is increasing with the widespread use of ultrasound (US) (1). Thyroid US is a widely accepted imaging modality for the initial assessment of thyroid nodules. It has been widely used to stratify the risk of malignancy in thyroid nodules and also in aiding with making decisions about whether fine-needle aspiration (FNA) is indicated. There are well-established ultrasound findings that differentiate benign and malignant thyroid nodules (2–8). A study by Kim and cols. (7) previously reported that hypoechogenicity, marked hypoechogenicity, microlobulated or irregular margins, microcalcifications, and taller than wide shape are the ultrasound features which best predicted the chance of malignancy in thyroid nodules. Since the malignancy risk estimated by US is not determined by a single US predictor, it should be assessed by a combination of the US features (9–11). There are several classification systems which categorize thyroid nodules according to the risk of cancer (12–21). An interesting thyroid imaging reporting and data system (TI-RADS) derived from the breast imaging reporting and data system (BI-RADS) was prospectively tested in 4550 nodules where it demonstrated a high sensitivity and NPV for the diagnosis of thyroid carcinoma (21). One of the limitations of this recent version of TIRADS was related to some significant US signs not considered for the flow chart, such as the halo sign, size and central flow by Doppler study. This way, the American Thyroid Association's (ATA) thyroid nodule guideline (20) proposed a new ultrasonographic pattern considering nodule margins. There has been no standardized malignancy risk stratification system for thyroid nodules. Thus, it is important to validate these classifications in different healthcare centers. We proposed to evaluate the diagnostic accuracy of a modified TI-RADS and 2015 ATA's ultrasound risk for the diagnosis of malignancy in thyroid nodules.

## MATERIALS AND METHODS

### Patients

Between July 2014 and August 2015, we prospectively analyzed data from 178 consecutive unselected patients with thyroid nodules attending the Endocrinology Division at *Santa Casa de Misericórdia de Porto Alegre*, a tertiary, university-based hospital located in an iodine-replete area in Southern Brazil. All patients underwent a complete clinical evaluation and thyroid ultrasonography. Patients with known thyroid cancer and/or patients with purely cystic nodules were excluded. This study was approved by the local ethics committees and participants provided written informed consent (CAAE:16398613.2.0000.5335).

### Imaging technique and TI-RADS and ATA ultrasound classification

**Figure 1:**
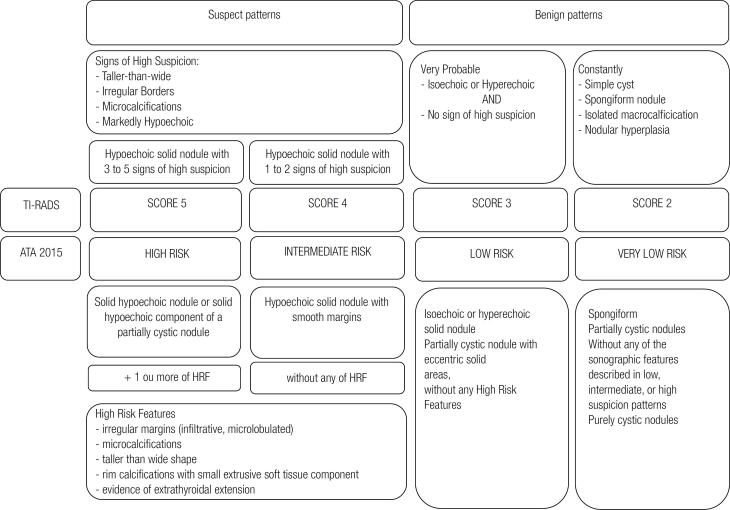
Comparative chart: TI-RADS (modified from Russ and cols.) and American Thyroid Association (ATA) 2015 HRF: High Risk Features.

**Figure 2:**
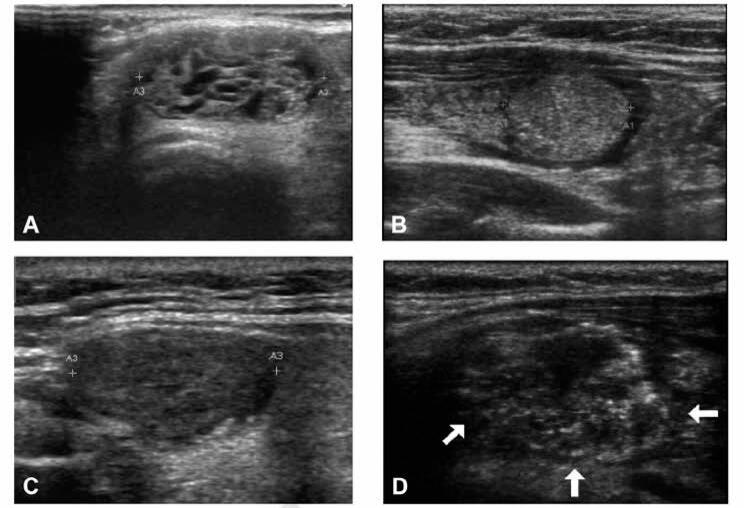
Representative images of TI-RADS and ATA systems in thyroid nodules. **A:** Spongiform nodule – TI-RADS 2 or ATA very low risk. **B:** Isoechoic solid nodule, regular-shaped and borders, without HRF - TI-RADS 3 or ATA low risk. **C:** Hypoechoic solid nodule with regular borders – TI-RADS 4 or ATA intermediate risk. **D:** Hypoechoic solid nodule with irregular borders and microcalcifications (arrows) – TI-RADS 5 or ATA high risk.

Thyroid Ultrasound Conventional B-mode and Doppler images of the neck and thyroid gland were obtained by ultrasound machine (ACUSON S2000^™^, Siemens and ACUSON Antares^™^, Siemens HealthCare, Erlangen, Germany) using a high-frequency probe (12 MHz). All US examinations were performed by the same radiologist (RFI) who has more than 10 years of experience in thyroid ultrasound. All images were examined on realtime two-dimensional gray-scale and Doppler imaging. All sonograms obtained were saved in a picture archive. Ultrasound features were assessed for each nodule characteristic like composition (solid, cystic, mixed), echogenicity (hyperechoic, isoechoic, hypoechoic, markedly hypoechoic), margins (well defined with or without halo sign, microlobulated, ill-defined, irregular), presence of calcification (microcalcification, macrocalcification), and shape of the nodule (round, oval). Also, the presence of cervical lymphadenopathy was evaluated. Findings that were considered in favor of a malignancy were hypoechoic or markedly hypoechoic in echogenicity; irregular, microlobulated, or ill-defined margins; presence of microcalcification; round shape and the presence of lymphadenopathy‥ We performed a prospective evaluation using the modified Russ classification (21), each nodule was classified into a TI-RADS category (2, 3, 4 and 5) based on the US features (Figure 1). Differently from the Russ classification (21) in which mildly or moderately hypoechoic nodules (TI-RADS 4A) are categorized differently from markedly hypoechoic nodules (TI-RADS 4B), we have decided not to subdivide category 4 with the intention of simplifying this score for clinical practice. Posteriorly, the same radiologist (RFI) who was blind about the pathological results, scored all evaluated nodules of the saved pictures using a flowchart (Figure 1) based on new ATA thyroid nodule guideline of as previously published (20). Based on the number of features suspicious for malignancy we considered four different sonographic patterns: “very low suspicion”; “low suspicion”; “intermediate”; and “high suspicion”. Pure cystic nodules were not included in the analyses. Figure 2 demonstrate representative US features in thyroid nodules.

The diagnostic performance of TI-RADS and ATA classification system was evaluated by comparison with the fine-needle aspiration cytology (FNA) reports and anatomopathological examination.

### Thyroid FNA, thyroid cytology, and histology

All 195 nodules were submitted to FNA performed by using a capillary US-guided (FNA-US) technique with 23-gauge needle attached to a 10 mL disposable plastic syringe. There was no nodule size threshold for indicating FNA. In most of the cases, only one needle pass was made per lesion. Cytology smears were prepared on four to six slides. Slides were fixed immediately in 95% alcohol and stained with Papanicolaou stain. One cytopathologist from of our institution who has vast experience in thyroid pathology interpreted the smears. A thyroid FNA specimen was considered satisfactory if at least 6 groups of follicular cells were present, and each group comprised at least 10 cells (22). The Bethesda System for Cytological Classification of Thyroid Nodules was used to interpret smears (23) as: 1) non-diagnostic or unsatisfactory, 2) benign, 3) atypia of undetermined significance, 4) a follicular neoplasm or suspicious for a follicular neoplasm, 5) suspicious for malignancy, and 6) malignant. Surgery was indicated based on cytopathological results (Bethesda 4, 5 and 6), or when the nodule was benign (Bethesda 2) but larger than 3-4 cm and causing compressive symptoms.

Anatomopathological examinations of tissue samples obtained at thyroidectomy were carried out according to the World Health Organization Guidelines (24), and the pathology reports pertaining to these samples were considered identical to the gold standard for the diagnosis of thyroid cancer.

### Statistical analysis

Clinical, laboratory, ultrasonography and cytological data, which are reported as the mean – standard deviation (SD) values, or as the median with percentiles between 25 and 75 (continuous variables), or as absolute numbers and percentages (categorical variables), were compared using Mann-Whitney U-test or chi-squared test as appropriate. Specificity, sensitivity, positive and negative predictive value were calculated to evaluate the reliability of TI-RADS and ATA classification methods in differentiation between benign and malignant features. In all analyses,. *P*. < 0.05 was considered for statistical significance. Statistical analysis of the results was performed with SPSS software (Statistical Package for Social Sciences) version 18.0.

## RESULTS

### Demographic data and global results of 195 nodules by US features and scores

The clinical characteristics of the 178 patients (195 nodules) included in this study were as follow: the median age was 59 years (range 49-66) and 94.9% were female. The median size of nodules was 24 mm (range 15-37). Nodules were classified as TI-RADS 2, 3, 4 and 5 in 11, 43, 44, and 2% of cases, respectively. Posteriorly, nodules were re-classified by ATA scores as very low risk in 35.9%; low risk in 28.2%; intermediate in 30.8% and high in 5.1% of cases. There was no difference in the size of the nodules among the TIRADS scores as well as among the ATA sonographic categories (p = 0.25 and 0.20, respectively).

The cytological descriptive statistics results are as follow: 15.9% (n = 31) nondiagnostic (Bethesda 1), 68.2% (n = 133) benign (Bethesda 2), 4.3% (n = 9) atypia of undetermined significance (Bethesda 3), 6.7% (n = 13); suspicious for a follicular neoplasm (Bethesda 4), 2.1% (n = 4) suspicious for malignancy (Bethesda 5) and 2.6% (n = 5) malignant (Bethesda 6).

Final histopathological results were available for 45 cases after surgery. Surgery was indicated based on cytopathological results (5 malignant, 4 suspect, 8 follicular neoplasm, and 3 atypia of indeterminate significance cases) or when the nodule was benign but larger than 3 cm and causing compressive symptoms (25 cases). There were 35 benign cases: 18 adenomatous goiters, and 17 adenomas. There were 10 malignant cases (5.13%), 9 classical papillary thyroid carcinomas, and 1 follicular thyroid variants of papillary carcinoma.

### Diagnostic performance of TI-RADS and ATA scores compared with cytological results

By cytology, 77% of TI-RADS scores 2 and 3 were benign (Bethesda category 2), 8.6% were indeterminate (Bethesda categories 3 and 4), and 1.9% were suspicious of malignancy (Bethesda categories 5) and none were malignant (Bethesda 6). For ATA score, 79% of low and very low risk were benign; 8.9% were indeterminate, 0.8% were suspicious of malignancy and none were malignant. Interestingly, 100% of carcinomas (Bethesda 6) and 50% of suspicious lesions (Bethesda 5) were classified as TI-RADS scores 4 and 5, and 100% of carcinomas and 75% of suspicious lesions were classified as intermediate and high risk ATA score.

To compare TI-RADS and ATA score with cytological results, only Bethesda categories 2 and 6 were used (n = 138), as the probability of mistake of these two categories is < 3%. The sensitivity, specificity, NPV, and accuracy of the TI-RADS were 100, 61.6, 100, and 63% respectively. In same way, the sensitivity, specificity, NPV, and accuracy of the ATA score were 100, 75, 100, and 76% respectively. The estimated pretest probability of malignant nodule was at 3.6% for the cytology endpoint.

### Diagnostic performance of TI-RADS and ATA scores compared with histopathological results (n = 45)

Distribution of carcinomas among TI-RADS categories 2, 3, 4 and 5 was 0, 5.5, 26 and 100%, respectively (Table 1). Among ATA score the percentage was 0, 0, 28, and 83% for “very low”, “low”, “intermediate suspicion” and “high suspicion”, respectively (Table 2). When compared to histopathological results, sensitivity, specificity, NPV, and accuracy of the TI-RADS it was 90, 51.4, 94.7, and 60%, respectively. In addition, the sensitivity, specificity, NPV, and accuracy of the ATA score was 100, 60, 100, and 68%, respectively. The estimated pretest probability of malignant nodule was at 22.2% for the histology endpoint. The matched results of TI-RADS and ATA categories with final histopathological and cytological results are shown in Table 3.

**Table 1:** TIRADS categories and risk of malignancy by final histopathological

TIRADS category	Benign	Malignant	Total	Risk of malignancy (%)
TIRADS 2 (n= 2)	1	0	1	0
TIRADS 3 (n= 18)	17	1	18	5.5
TIRADS 4 (n= 23)	17	6	23	26
TIRADS 5 (n= 3)	0	3	0	100
Total (n= 45)	35	10	45	

**Table 2:** ATA categories and risk of malignancy by final histopathological

ATA category	Benign	Malignant	Total	Risk of malignancy (%)
High suspicion (n= 6)	1	5	6	83.3
Intermediate suspicion (n= 18)	13	5	18	27.7
Low suspicion (n= 10)	10	0	10	0
Very low suspicion (n= 11)	11	0	11	0
Total	35	10	45	

**Table 3:** Matched results of TIRADS and ATA categories with final histopathological and cytological results

Ultrassonographic score	Benign histological results (N = 35)						Malignant histological results (N = 10)					
	I	II	III	IV	V	VI	I	II	III	IV	V	VI
TIRADS 2	0	1	0	0	0	0	0	0	0	0	0	0
TIRADS 3	0	12	2	2	1	0	0	0	0	0	1	0
TIRADS 4	4	5	3	4	1	0	0	1	0	2	0	3
TIRADS 5	0	0	0	0	0	0	0	0	0	0	1	2
												
ATA VERY LOW RISK	0	7	1	2	1	0	0	0	0	0	0	0
ATA LOW RISK	0	7	1	2	0	0	0	0	0	0	0	0
ATA INTERMEDIATE RISK	4	4	3	1	1	0	0	1	0	2	0	2
ATA HIGH RISK	0	0	0	1	0	0	0	0	0	0	2	3

### Reclassification of thyroid nodules from TI-RADS to ATA score

Twenty-one nodules TI-RADS category 4 were reclassified according to ATA score as very low risk (10 cases) and low risk (11 cases). Of the ATA very low risk cases, 8 presented cytological results of Bethesda 2 (benign) and 2 cases were Bethesda 3, one of them being submitted to surgery and the histopathological diagnosis was follicular adenoma. Of the ATA low risk cases, 10 were classified by cytological analysis as Bethesda 2. One case was Bethesda category 3 and the outcome of the anatomopathological was follicular adenoma.

### Performance of TI-RADS and ATA scores in thyroid nodules with indeterminate results on cytology

Of the 12 indeterminate nodules (Bethesda categories 3 and 4) examined, 10 (83.3%) were histologically benign. Sonographic classification of nodules by TI-RADS category 2 or 3, or as very low to low suspicion by ATA standards displayed negative predictive value of 100% for both systems. Positive predictive values for TI-RADS categories 4 and 5 and ATA intermediate and high risks were 54.5 and 40%, respectively.

## DISCUSSION

The ultrasonography terminology of thyroid nodules should be feasible for clinical application, should be useful for malignancy risk stratification, and show a low inter observer variability. Here, we demonstrated a very high NPV and a high sensibility to cancer diagnosis of scores TI-RADS and ATA ultrasound risk.

The Thyroid Imaging Reporting and Data System (TI-RADS) was used by Park and cols. (12) and Horvath and cols. (13) and both systems appear to be difficult to use in routine clinical practice. In order to achieve a practical tool for analyzing thyroid nodules and to improve communication between radiologists and physicians, Russ and cols. (21) proposed a new TI-RADS classification that has a high sensitivity (95.7%) and NPV (99.7%) for diagnosis of thyroid carcinoma. Accordingly, we found NPV of 94.7% for scores TI-RADS 2 and 3.

Recently, the American Thyroid Association (ATA) proposed a classification of thyroid nodules into five categories based on US features (20). In the same way, the reassessment of ATA ultrasound classification of malignancy in our patients shows a very high NPV (100%) for very low and low ATA ultrasound risk. These results confirm a high probability of both systems to discard malignancy. In fact, TI-RADS 2 or 3, and ATA very low or low risk consider similar benign patterns like spongiform nodules, isoechoic or hyperechoic solid nodules without signs of high suspicion. Interestingly, isoechoic solid nodules with some suspicious finding are not included in any of the TI-RADS or ATA categories. The risk of malignancy found in previous studies was between 16 to 20%, similar to that observed for hypoechoic nodules without any suspicious finding (25–27). We observed a prevalence of malignancy of 5.5% of nodules categorized as TI-RADS 3, similarly observed by Russ and cols. (4.3%) (21). Also, no nodule classified as low suspicion by ATA was malignant in our sample, reinforcing data from Rosario and cols. (25) that demonstrated in a large number of nodules the risk of malignancy of only 1.7%, lower than suggested by ATA (5-10%) in this category. TI-RADS category 2 or 3 and ATA very low and low risk represented a significant proportion of patients, 54% and 64%, respectively. Therefore, proven high sensitivity and NPV of both systems could allow to ultrasonographically (without FNA) monitoring these nodules categories, especially nodules under 2 cm, unless they increase in volume or if there are new suspicious sonographic features.

TI-RADS proposed by Russ (21) categorize differently mild or moderate hypoechoic nodules (TI-RADS 4A) from markedly hypoechoic nodules (TI-RADS 4B) and unlike this system, we did not subdivide the category 4 of TI-RADS considering this category hypoechoic solid thyroid nodules. This was done to simplify the TI-RADS system for clinical practice minimizing discrepancies in the evaluation of the degree of hypoechogenicity. Also, to calculate the accuracy of TIRADS, it was necessary to group these categories. In addition, there is a general recommendation of US-PAAF of hypoechoic thyroid nodule especially above 1 cm, regardless of the degree of hypoechogenicity (20,28). Thus, TI-RADS 4 nodules were considered an intermediate suspicious for malignancy and were evaluated by cytology. In contrast to TI-RADS, hypoechogenicity associated with only one suspicious finding is sufficient for a nodule to be classified as “high suspicion” by ATA (20). Only 5.8% (5 of 86) of our TI-RADS 4 were reclassified to ATA “high suspicion”, meaning that most of TI-RADS 4 nodules were hypoechoic solid thyroid nodules without sonographic patterns of high suspicion, considered by ATA as “intermediate suspicion”. We believe this is due to a better definition of the nodule margins (smooth *vs* irregular margins) proposed by ATA (20). In addition, we found a similar prevalence of malignancy of 26% for TI-RADS 4 and 28% for ATA intermediate suspicion. In fact, the risk of malignancy estimated by ATA in the “intermediate suspicion” category is 10 to 20% and, unlike Rosario that demonstrated only 9.9% for intermediate suspicion, we found a significant frequency of carcinoma in this category.

We observed a malignancy risk of 100% of nodules TI-RADS 5. In fact, this category was composed of highly suspect nodules with more than 3 signs of high suspicion. For ATA “high risk” that included hypoechoic nodules with one or more signs of high suspicion, we found 83% of carcinomas, similar to risk estimated by ATA of 70-90% and a higher rate than what was observed by Rosario (54.8%) (25). Previous results of same author have been demonstrated that “high suspicion” category of ATA, markedly hypoechoic nodules with one or more suspicious findings and mildly or moderately hypoechoic with two or more suspicious findings had a similar risk of malignancy of 66% (95 % CI 57.6-73.7%) (25). These results reinforce that the degree of hypoechogenicity of nodules may not be so definitive in determining the suspicion pattern as the association of established features predictive of malignancy.

In our study, US reevaluation had some disagreements regarding nodules mildly hypoechoic without suspicious US features classified initially as TI-RADS 4 that were reclassified as very low or low by ATA risk. This could have happened due to the heterogeneity of the nodules (hypoechoic areas between iso- hyperechoic areas) and the characteristics of borders, a feature not well defined in TI-RADS score. Moreover, it is important to note that poorly defined margins are sometimes difficult to delineate, and are not equivalent to irregular margins. An irregular margin, which indicates the demarcation between nodule and parenchyma, is clearly visible but demonstrates an irregular, infiltrative or spiculated course‥ The reassessing of these nodules in a low suspicion category by ATA was strongly corroborated by cytology and/or histology confirming benign thyroid lesions.

The TI-RADS classification does not consider nodular size to indicate FNA-US (21). However, ATA risk score has recommended size threshold for biopsy (no biopsy in benign, > 2 cm in very low, > 1.5 cm in low, > 1 cm in intermediate, and > 1 cm in high risk, respectively) (20). In fact, the correlation between the nodule size and the risk for malignancy remains controversial. Although a recent systematic review suggested that the larger nodules present a higher pretest probability of malignancy (29). In addition, the growth of a nodule is not a reliable predictor of malignancy since many benign nodules can slowly grow over time (30–33). In our study, no correlation was found between the size of the nodules and sonographic risk assessments, reinforcing the importance of ultrasound findings more than the size of thyroid nodule in indicating FNAB-US. However, the increase of incidence of thyroid cancer in the last years, due to the diagnosis of microcarcinoma (25%), supports the ATA recommendation (20).

Even with few samples evaluated by histology, a good performance of TI-RADS and ATA ultrasound risk was observed in thyroid nodules with indeterminate cytology (Bethesda categories 3 and 4). Sonographic classification of nodules by TI-RADS category 2 or 3, or as very low to low suspicion by ATA displayed NPV of 100% for both systems. Positive predictive values for TI-RADS category 4 and 5 and ATA intermediate and high risk rose the cancer prevalence to 54.5 and 40%, respectively, compared to described prevalence of 5-15% (0% in our sample) of Bethesda 3 nodules and 15-30% (15.4% in our sample) for Bethesda 4. Previous studies have demonstrated that TI-RADS 3 and 4A scores led to 80% sensitivity and 90% NPV in Bethesda 3 cases. In contrast, for nodules scored as TI-RADS 4B and 5, the combined cytological results of Bethesda 4 and 5 resulted in a higher risk of malignancy (75% and 76 9%, respectively, P < 0.001) (34). More recently studies confirm a NPV values for ATA and TI-RADS were 91 and 74%, respectively, to rule out malignancy in cytologically indeterminate thyroid nodules (35). Further, taken together, these results suggest that cytological indeterminate nodules with TI-RADS 2 or 3 and ATA very low or low risk sonographic pattern have a higher likelihood of being benign.

This study has some limitations because we retrospectively evaluated thyroid nodules by ATA system. Also an inter-observer analysis was not possible, as only one radiologist with thyroid expertise evaluated the patients. The small number of histopathology examinations of nodules with indeterminate cytology, limited conclusions of this subgroup. In addition, this study represents a single specialized thyroid clinic's work with the intent to implement TI-RADS and/or ATA diagnostic guidelines in our clinical practice; however, results have to be confirmed by other centers.

In conclusion, the sonographic patterns proposed by TI-RADS and the recently revised ATA's guidelines have high sensitivity and NPV for the diagnosis of thyroid carcinoma. Both systems are feasible for clinical application and have an excellent negative predictive value allowing the selection of patients to FNA-US.
